# Incised Wound of Abdomen, with Wound and Protrusion of Intestine

**Published:** 1866-06

**Authors:** R. G. Jennings

**Affiliations:** Little Rock, Ark.; U. S. A.


*Incised Wound of Abdomen, with Wound and Protrusion of Intestine.* By A. A. Surg. R. G. Jennings, U. S. A.

Private Harry Jacobs, Co. L, U. S. Cavalry, aged 24 years, a native of Pennsylvania, was stabbed in the abdomen at 11 o'clock A. M., February 20th, 1866, with a small blade of a pocket knife. He stated that he was standing at the time, and that he felt something like a kick with an unusual degree of warmth over the bowels, followed by faintness, causing him to seek his bunk, which he reached, though seven or eight feet in height. Upon opening his pants he found his bowels protruded. I was instantly sent for, but being about half a mile distant, did not reach him for some time. The man was stretched upon his bunk, his knees drawn up, pulse feeble, the surface bathed in a clammy perspiration. There were three coils of intestines upon the parietes of the abdomen, which were estimated to be not less than three feet in length. I had considerable difficulty in finding the opening into the cavity of the abdomen; and, after finding it, could not reduce the protrusion without assistance. After placing the man on a table, I discovered the wound to be about midway between the centre of the left Poupart's ligament and the umbilicus, extending upward and outward. I ordered cloths wet in warm water to be applied over the bowels, and sent for medical assistance and instruments. A. A. Surg. E. V. Deuell, U. S. A., in charge of St. John's Hospital, arrived soon after. The patient was brought under the influence of chloroform, when the wound was enlarged half an inch in its original direction by means of my finger, a grooved director, and a sharp-pointed curved bistoury. I retained my finger in the wound, elevating the walls of the abdomen while Dr. Deuell, by manipulation, returned the bowels to their place within the cavity. When this was nearly accomplished, we discovered a wound in the intestine fully three-quarters of an inch in length, which was serrated and bled freely, although no fœcal matter escaped. In this wound two interrupted sutures were taken, the ends of the thread being clipped close to the knot. The edges of the wound in the abdomen were nicely adapted and retained with three deep interrupted sutures, not including the peritoneal membrane. A wet compress was now applied to the wound, and retained in its place by a broad bandage around the body. After recovery from the effects of chloroform, free emesis ensued, which would undoubtedly have broken the sutures in the abdomen had it not been firmly supported with both hands. I directed a nurse to be kept in readiness to render like assistance whenever necessary. A half grain of morphine was now given and the patient carefully conveyed to St. John's Hospital, where he came under the personal care of A. A. Surg. J. A. Dibrell. During the first twenty-four hours, and until after complete reaction was established, there was no medical interference. As symptoms of local peritonitis began to appear, cold water applications were kept constantly to the wounded part, and depressing doses of tr. aconite rad. and morphine were given him. This treatment was continued for several days, the doses being increased according to indications. On the fourth day an emollient enema was given with happy effects. From this time the wound rapidly improved; at the date of this report, it is entirely healed.

The soldier now expresses himself fully able for duty, and desires to go to his regiment. His bowels are regular and appetite good.

Little Rock, Ark., March 30th, 1866.

